# Induction of Ferroptotic Cell Death by Neuromelanin Pigments in Dopaminergic Cells

**DOI:** 10.1021/acschemneuro.5c00029

**Published:** 2025-03-27

**Authors:** Gizem Kaftan Öcal, Güliz Armagan

**Affiliations:** † Biochemistry PhD Programme, Graduate School of Health Sciences, Ege University, Izmir 35100, Türkiye; ‡ Department of Biochemistry, Faculty of Pharmacy, Ayfonkarahisar Health Sciences University, Afyonkarahisar 03218, Türkiye; § Department of Biochemistry, Faculty of Pharmacy, Ege University, Izmir 35100, Türkiye

**Keywords:** neuromelanin, ferroptosis, iron metabolism, GSH-GPX4 axis, neurodegeneration

## Abstract

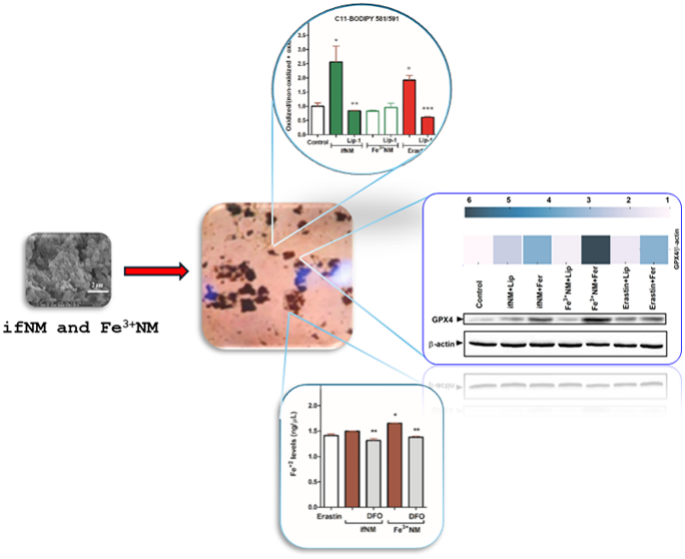

Neuromelanin (NM) is an iron-rich, insoluble brown or black pigment that exhibits protective properties. However, its accumulation over time may render it a source of free radicals. In Parkinson’s disease, dopaminergic neurons with the highest NM levels and increased iron content are preferentially vulnerable to degeneration. Considering NM’s iron binding capacity and the critical role of iron in ferroptosis, we aimed to investigate the interplay between neuromelanin and ferroptosis in dopaminergic cells. We prepared two NM pigments: iron-free NM (ifNM) and iron-containing NM (Fe^3+^NM) and, exposed to cells. After verifying NM accumulation, cell viability was assessed in the absence or presence of antioxidants (NAC (1 mM), Trolox (100 μM)) and specific inhibitors of cell death types. Ferroptosis-related parameters, including lipid peroxidation byproducts (4-HNE), lipid ROS, glutathione, intracellular iron, GPX4, and ACSL4, and cellular iron metabolism-related proteins (TfR1, ferroportin, ferritin, IREB2) were evaluated following ifNM and Fe^3+^NM treatments, with or without Ferrostatin-1, Liproxstatin-1 and deferoxamine. Both NMs induced cell death via distinct mechanisms. Ferroptotic cell death by ifNM and Fe^3+^NM was reversed by ferrostatin-1 and NAC (*p* < 0.05). Significant alterations in lipid peroxidation, GPX4 levels, and iron metabolism were observed independent of NM’s iron composition (*p* < 0.05). Ferritin levels increased following ifNM treatment, reflecting an adaptive response to iron overload, while Fe^3+^NM treatment led to ferritin depletion, possibly via ferritinophagy. Our findings reveal a distinct role of iron-rich and iron-free neuromelanin in modulating ferroptotic pathways, highlighting the potential of targeting neuromelanin-iron interactions as a therapeutic strategy to mitigate neuronal ferroptosis in Parkinson’s disease.

## Introduction

Neuromelanin (NM) is a dark colored, insoluble granular pigment primarily found in the dopaminergic and noradrenergic neurons of the substantia nigra pars compacta (SNpc) and locus coeruleus (LC) in the brain.^1^ Histological studies have shown that NM granules are located in the neuronal perikaryon and surrounded by a double membrane.^2^ Structurally, NM consists of melanic, aliphatic and peptide residues^1^ and is composed primarily of dopamine (DA) oxidation products, proteins, and lipids. Although the exact process of NM formation in cells remains unclear, it has been suggested that both enzymatic and nonenzymatic dopamine oxidation contribute to NM formation.^3^ Changes in NM levels as well as its intracellular or extracellular localization define its toxic or protective role under cellular conditions.^3,4^ While NM is traditionally considered neuroprotective due to its ability to chelate potentially toxic metals, including iron, emerging evidence suggests that excessive NM accumulation and iron dysregulation may contribute to neurodegeneration, particularly in Parkinson’s disease (PD).^5,6^ Dopamine oxidative products are known to induce mitochondrial dysfunction, impaired protein degradation and, α-synuclein aggregation into neurotoxic oligomers. Additionally, NM has been suggested to damage dopaminergic neurons by directly inhibiting proteasome function and catalyzing the production of free radicals by reacting with hydrogen peroxide.^7,8^

Parkinson’s Disease (PD) is the second most common neurodegenerative disease after Alzheimer’s Disease.^9^ Dopamine deficiency occurs as a result of progressive degeneration of dopaminergic neurons in the SNPc region of the brain. In addition to dopaminergic neuronal death, several mechanisms such as intracellular α-synuclein accumulation, oxidative stress, and iron accumulation are linked to PD pathophysiology. Iron acts as a cofactor in several metabolic processes; however, its homeostasis is tightly regulated due to its capacity to catalyze Fenton reactions, leading to the production of reactive oxygen species (ROS).^10^ Its accumulation in the brain increases with age and is more prominent in regions associated with neurodegenerative disorders.^11,12^ Altered iron levels are particularly observed in glial cells and dopaminergic neurons of SNpc, where iron levels have been found to correlate with disease severity.^13^

Ferroptosis, an iron-dependent regulated cell death pathway, is driven by lipid peroxidation, reduction in GPX4 enzyme activity, and iron overload.^14^ Disruption in iron metabolism and an increase in ferroptosis markers have been implicated in the pathogenesis of several neurodegenerative disorders, including PD.^15^ The presence of iron in the formation of lipid-derived reactive oxygen species (ROS)—particularly lipid hydroperoxide—via the Fenton reaction or iron-dependent oxidases, is one of the primary mechanisms of ferroptosis.^16^

In Parkinson’s disease, dopaminergic neurons containing NM are particularly vulnerable to degeneration with its degradation products that can trigger microglial activation and inflammatory responses.^7,17^ Paradoxically, NM protects neurons by sequestering excess iron, inhibiting Fenton reaction and preventing the initiation of ferroptosis.^18,19^ This dual role underscores NM’s critical involvement in neuronal health and ferroptosis-related pathways. In this study, we aimed to explore how NM’s iron-binding properties influence ferroptosis susceptibility and whether NM accumulation serves as a protective or detrimental factor in neurodegeneration. By elucidating these mechanisms, we aim to shed light on potential therapeutic targets for mitigating ferroptosis-mediated neuronal damage in Parkinson’s disease and related neurodegenerative disorders.

## Results and Discussion

### SEM Imaging of ifNM and Fe^3+^NM

**Figure 1:**
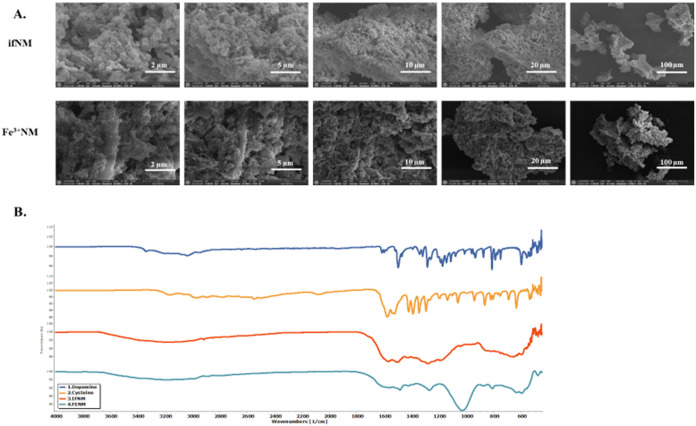
SEM images of ifNM and Fe^3+^NM. The image is 6.75 and 4.50 m (A). IR spectra of dopamine, cysteine and NMs (ifNM and Fe^3+^NM) (B).

In this study, we synthesized and characterized two NM types: iron-free NM (ifNM) and iron-containing NM (Fe^3+^NM), allowing us to dissect the specific contribution of iron composition in NM to ferroptotic pathways, shedding light on the interplay between NM, iron homeostasis, and lipid peroxidation in dopaminergic cells. ifNM and Fe^3+^NM samples were visualized using scanning electron microscopy (SEM) (Figure 1A). SEM imaging revealed that NM granules exhibit a multilayered, graphite-like structure, with large aggregates (∼3–4 μm) forming through the combination of smaller spherical particles, consistent with previous reports.^20^ FT-IR experiments were performed to evaluate and compare the structures of dopamine, cysteine, ifNM and Fe^3+^NM (Figure1B). Considering the IR spectra of dopamine; the NH, OH and C–O stretching bands were observed at 3338, 3202, 1284 cm^–1^ respectively while NH and OH bending bands were detected at 1616 and 1393 cm^–1^ respectively. The stretching bands of SH, C=O, C–O, NH and the bending bands for OH and NH groups were observed at 2550, 1576, 1295, 3170, 1421, 1525 cm^–1^ respectively which were confirmative for cysteine. In the IR spectra of ifNM; the C=O stretching bands and OH out of plane bending bands for carboxylic acid was shifted slightly to lower frequencies (1571 and 1427 cm^–1^) compared to the bands observed for cysteine. In Fe^3+^NM spectra, the C=O stretching bands for carboxylic acid was shifted slightly to lower frequencies (1561 cm^–1^) while OH out of plane bending bands moved to higher frequencies (1486 cm^–1^) compared to the bands observed for NM. In addition, ifNM and Fe^3+^NM were subjected to the IR analysis and their results were compared with the results of starting compounds dopamine and cysteine. The IR spectral analysis confirmed the successful synthesis of NM, with specific chemical shifts indicating iron-NM interactions, supporting earlier findings.^21,22^

### Intracellular Entry and Cytotoxic Effects of ifNM and Fe^3+^NM

**Figure 2:**
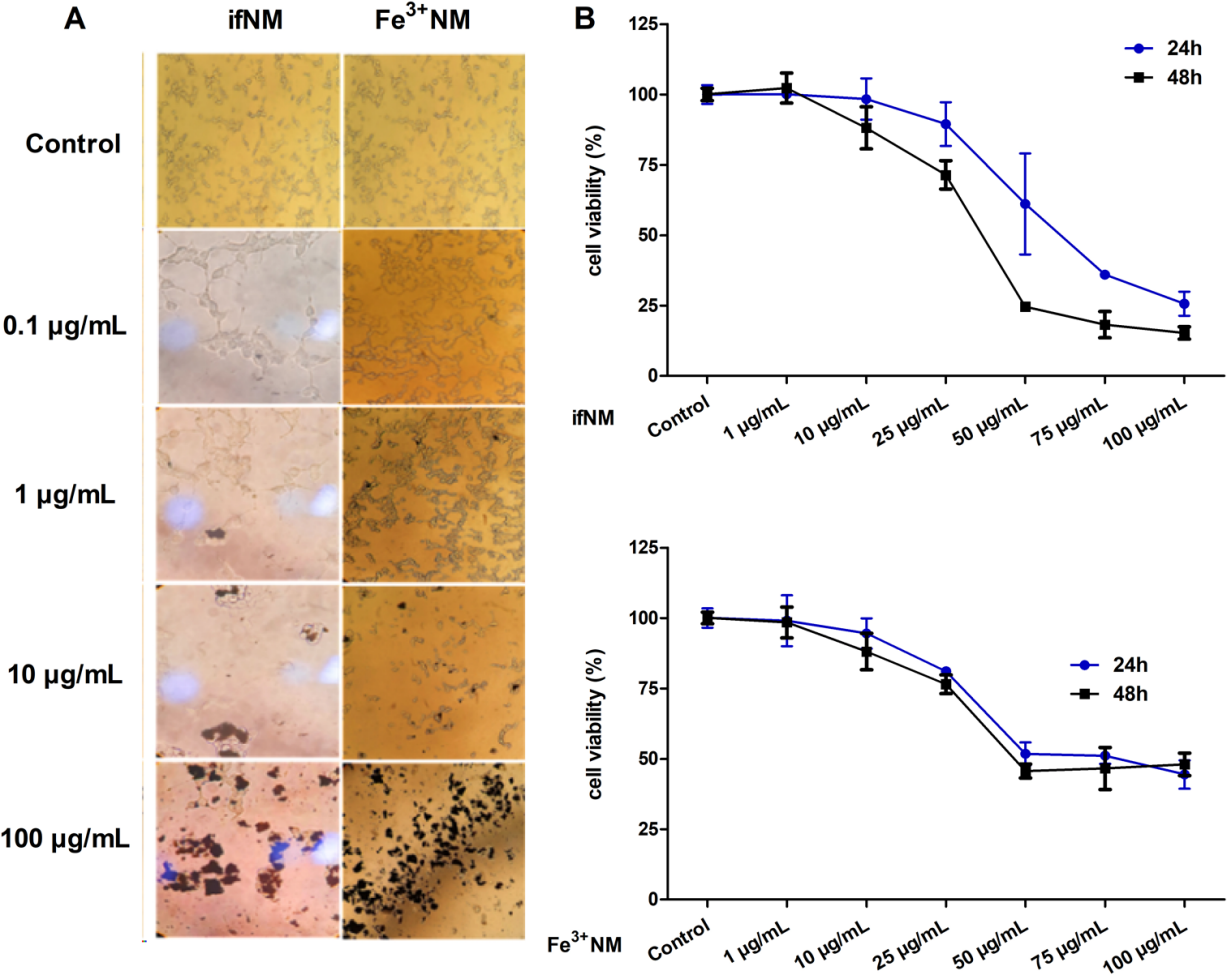
Light microscope images showing the uptake of NM pigments (ifNM and Fe^3+^NM) by SH-SY5Y cells after 48 h of treatment at concentrations of 0.1, 1, 10, and 100 μg/mL (A). Dose-dependent and time-dependent cell death after 24- and 48-h exposure to NMs (ifNM and Fe^3+^NM) at concentrations of 1, 10, 25, 50, 75, and 100 μg/mL (B). Cell viability was assessed using the WST-1 assay.

The entry of NMs (0.1, 1, 10, and 100 μg/mL) into the cell was visualized with a light microscope in SH-SY5Y cells (Figure 2A). The accumulation of NMs was observed clearly when NM pigments were applied at higher concentrations (10 and 100 μg/mL) at both time points. Although NM treatment at 1 μg/mL did not induce toxicity, the entry of the NMs observed in characteristic brown color. The effect of NM on cell viability was investigated. NMs were applied to cells at concentrations of 1, 10, 25, 50, 75, and 100 μg/mL for 24 and 48 h, and cell viability was evaluated by the WST-1 method (Figure 2B). The IC_50_ values for ifNM were 54.47 ± 6.11 μg/mL at 24 h and 30.68 ± 4.05 μg/mL at 48 h, while for Fe^3+^NM, the IC_50_ values were 30.26 ± 4.29 μg/mL at 24 h and 25.44 ± 4.46 μg/mL at 48 h. Viceconte et al. reported that the application of synthetic NM to BV2 cells significantly reduced cell viability.^17^ As observed in previous studies, NM treatments reduced dopaminergic cell viability in a concentration-dependent manner, demonstrating its cytotoxic effects.

### Effect of ifNM and Fe^3+^NM on LDH Release

**Figure 3:**
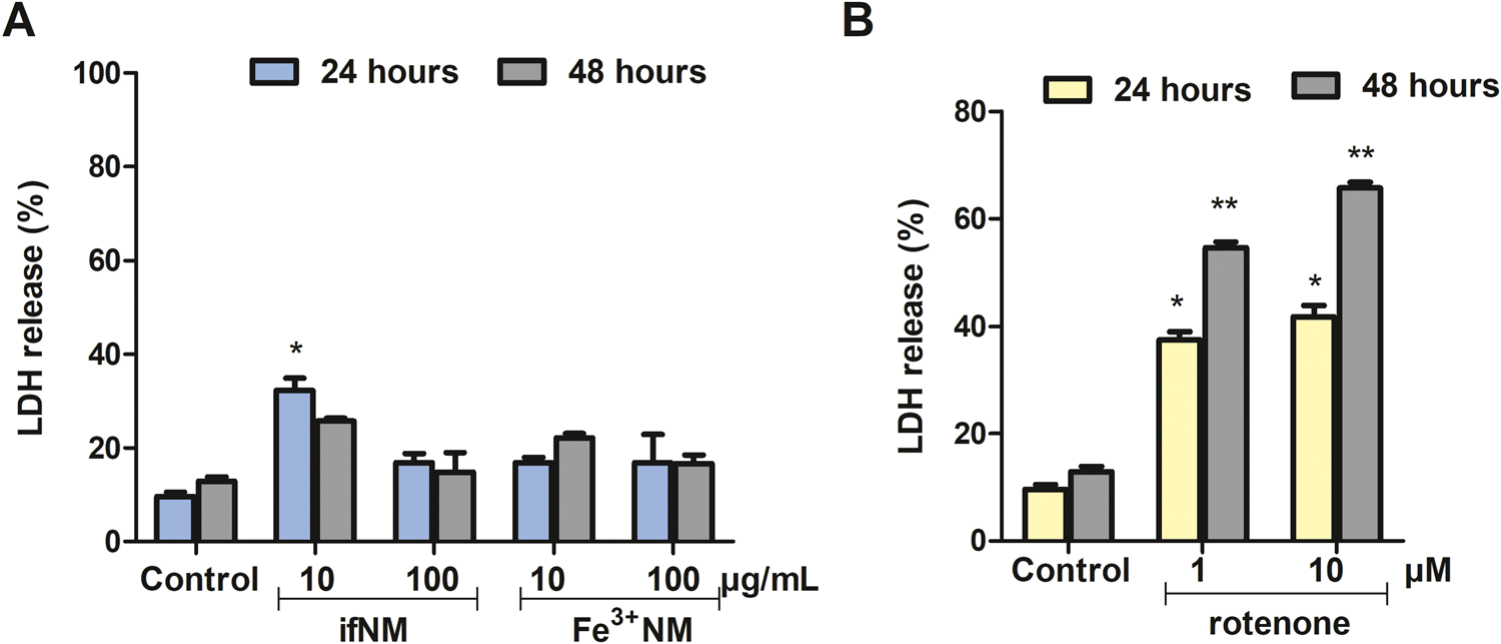
Changes in LDH release in SH-SY5Y cells following 24- and 48-h of exposure to ifNM and Fe^3+^NM at concentrations of 10 and 100 μg/mL (A). Rotenone (1 and 10 μM) was used as a cell death inducer (B). 10% Triton X-100-treated wells were used as maximum release (100%) wells. *p < 0.05; compared with vehicle control group.

As a marker of cell membrane disruption, LDH release was evaluated following the exposure of ifNM and Fe^3+^NM at 10 and 100 μg/mL concentrations. At 24 h, maximum LDH release was observed at concentration of 10 μg/mL for ifNM (*p* < 0.05). At 48 h, both agents displayed a concentration-independent plateau effect (Figure 3A). Prolonged exposure to ifNM and Fe^3+^NM resulted in a plateau effect, with LDH release stabilizing across concentrations, while Rotenone induced a pronounced cytotoxic response (Figure 3B). During ferroptosis, the accumulation of lipid peroxides compromises cell membrane integrity, leading to the release of intracellular contents, including LDH, into the extracellular space. This phenomenon has been observed in various studies where ferroptosis-inducing agents (e.g., Erastin, RSL3) increased LDH release, an effect that was prevented by ferroptosis inhibitors or the genetic knockdown of ferroptosis regulators.^23−25^ In our study, LDH release supported the notion that NM may exhibit cytotoxic effects on dopaminergic cells (Figure 3). However, ifNM and Fe^3+^NM treatments displayed distinct patterns of LDH release. While ifNM treatment at low concentration led to a marked increase in LDH release, suggesting membrane integrity loss possibly linked to necrotic-like cell death, Fe^3+^NM treatment exhibited minimal LDH release, even at higher concentrations. This observation implies that Fe^3+^NM primarily triggers ferroptosis, a regulated cell death pathway characterized by lipid peroxidation and intracellular oxidative damage, rather than extensive membrane disruption typically seen in necrosis. This is consistent with the fact that ifNM seems to be a reactive compound capable of directly interacting with membrane components, potentially leading to membrane damage and subsequent LDH release. In contrast, Fe^3+^NM’s effects appear to be primarily mediated through intracellular iron-dependent lipid peroxidation and oxidative damage, rather than direct membrane reactivity.

It is important to note that while LDH release serves as an indirect indicator of ferroptotic cell death, it is not a specific marker for ferroptosis, as other forms of cell death (e.g., necrosis, pyroptosis) can also cause LDH release. Therefore, LDH assays are most reliable when used in conjunction with other ferroptosis-specific markers, such as lipid ROS accumulation (e.g., C11-BODIPY staining), 4-HNE levels, GPX4 activity, and glutathione (GSH) levels.

### Effect of ifNM and Fe^3+^NM on Cell Viability in the Presence of Antioxidants

**Figure 4:**
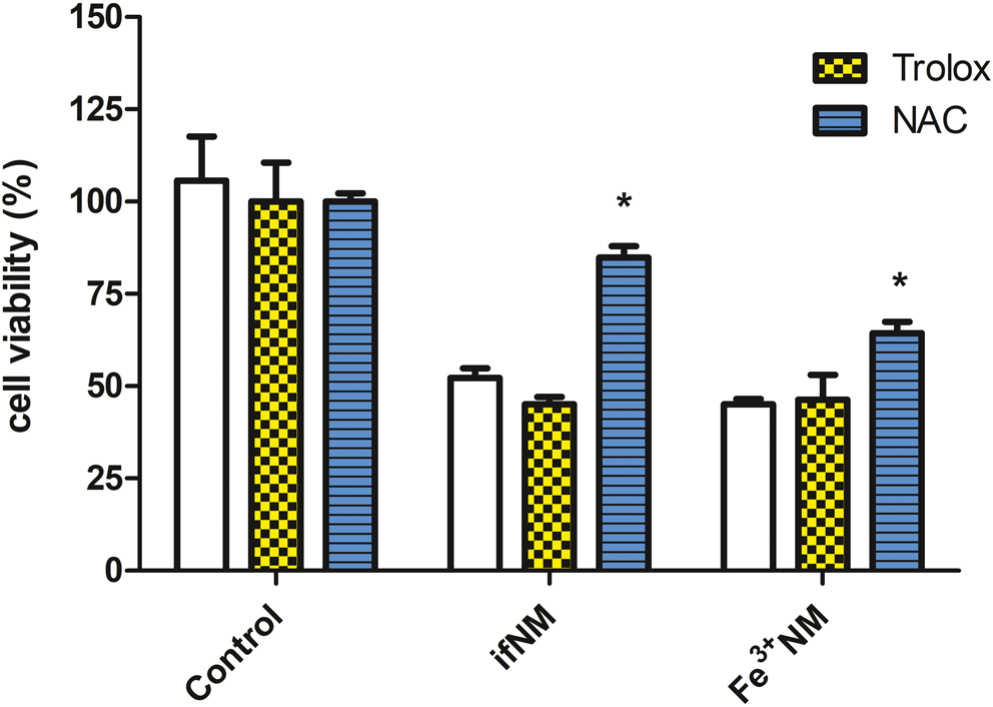
Protective effects of antioxidants (NAC, 1 mM; Trolox, 100 μM; 1-h pretreatment) on SH-SY5Y cells following 24-h exposure to ifNM and Fe^3+^NM at their respective IC_50_ values. The IC_50_ values for ifNM and Fe^3+^NM were 54.47 ± 6.11 and 30.26 ± 4.29 μg/mL, respectively. Cell viability was assessed using the WST-1 assay. *p < 0.05; compared with only ifNM- or only Fe^3+^ NM-treated cells.

The effect of ifNM and Fe^3+^NM on cell viability at their IC_50_ values was evaluated in the presence of antioxidants, NAC (1 mM) and Trolox (100 μM). Cell viability was higher in the NM + NAC-treated groups compared to NM-only-treated groups (84.8% for ifNM + NAC-treated cells and 64.4% for Fe^3+^NM + NAC-treated cells) (*p* < 0.05) (Figure 4). Cell death caused by ifNM and Fe^3+^NM was statistically reversed by NAC. On the other hand, Trolox had no effect in the NM-treated cells (Figure 4). NAC serves as a GSH precursor, providing cysteine essential for cellular antioxidant defense. By replenishing GSH levels, NAC supports the cell’s antioxidant system against oxidative damage. One of the key parameters in ferroptosis progression is the decrease in GPX4 activity, an essential enzyme for neutralizing lipid peroxides. In our study, NAC treatment significantly increased cell viability in both ifNM and Fe^3+^NM groups (Figure 4A). Studies have shown that NAC reduces lipid peroxidation by enhancing GPX4 expression, thereby reversing ferroptosis.^26^

### Effects of ifNM and Fe^3+^NM on Cell Death Types

**Figure 5:**
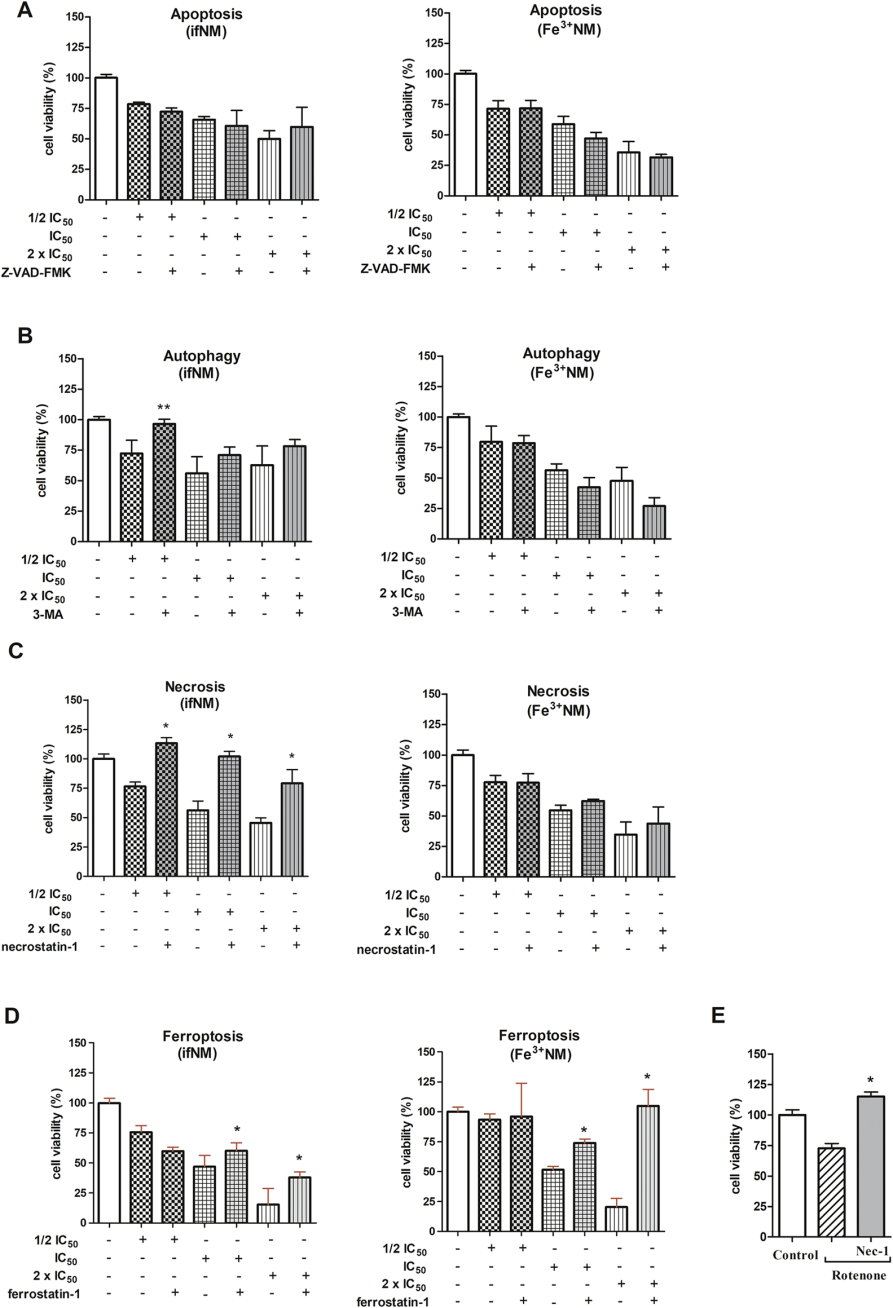
Percentage of cell viability in the presence of apoptosis (A), autophagy (B), necrosis (C) and ferroptosis (D) inhibitors following ifNM and Fe^3+^NM treatments. (E) Rotenone (10 μM) was used as a cell death inducer. Z-VAD-FMK (20 μM, 1 h), 3-MA (5 mM, 1 h), necrostatin-1 (50 μM, 1 h) and ferrostatin-1 (5 μM, 2 h) were applied to cells. Then, cells were incubated with ifNM and Fe^3+^NM (1/2 IC_50_, IC_50_ and 2 × IC_50_) for additional 24 h. The IC_50_ values for ifNM and Fe^3+^NM were 54.47 ± 6.11 and 30.26 ± 4.29 μg/mL, respectively. Cell viability was assessed using the WST-1 assay. *p < 0.05; compared with only NM-treated groups, **p < 0.01; compared with only NM-treated group.

The cytotoxic effects of ifNM and Fe^3+^NM were evaluated at three concentrations: 1/2 IC_50_, IC_50_ and 2 × IC_50_—by assessing different types of cell death. SH-SY5Y cells were pretreated for 2 h with inhibitors targeting apoptosis (Z-VAD-FMK, 20 μM), necrosis (necrostatin-1, 50 μM), autophagy (3-MA, 5 mM) and ferroptosis (ferrostatin-1, 5 μM). Accordingly, while ifNM and Fe^3+^NM did not affect the apoptotic pathway in cells, ifNM at three concentrations statistically induced necrosis (*p* < 0.05) (Figure 5A,C). In ferroptosis-evaluated groups, cell viability decreased in both ifNM- and Fe^3+^NM-only-treated groups compared to untreated cells. Notably, ferrostatin-1 pretreatment resulted in increased cell viability in the 2 × IC_50_ group of ifNM and in both the IC_50_ and 2 × IC_50_ groups of Fe^3+^NM. Accordingly, while ifNM was observed to trigger ferroptosis primarily at high concentrations, Fe^3+^NM effectively induced ferroptosis at both IC_50_ and 2 × IC_50_ concentrations. (Figure 5D).

The obtained data indicate that the presence or absence of iron in NM influences its cytotoxic effects. While ifNM was found to induce necrosis and ferroptosis at high concentrations, Fe^3+^NM predominantly caused ferroptotic cell death (Figure 5D). Furthermore, since cell death was not reversed in the presence of the pan-caspase inhibitor Z-VAD-FMK, it suggests that the synthesized NMs may not trigger apoptotic cell death.

In condition of excess iron ions, iron tends to accumulate in NM at different binding sites with varying affinities. NM and synthetic melanins contain both polynuclear high-affinity iron sites and mononuclear low-affinity iron sites. Under physiological conditions, iron preferentially binds to high-affinity sites, where it remains relatively stable. However, when these high-affinity sites become saturated, iron starts to accumulate in low-affinity sites, where it is more prone to mobilization and redox cycling, leading to the generation of free radicals and oxidative stress. Thus, accumulated iron can become redox-active, facilitating the generation of free radicals and contributing to neurotoxic consequences. Previous studies have reported that redox activity in NM aggregates from PD patients increases proportionally with iron content.^27^ Analysis of NM from SNpc of PD patients also revealed iron accumulation and excessive binding, potentially exacerbating oxidative stress.^2,28,29^ While the iron content in NM can initially protect cells from necrotic cell death by sequestering free iron, unbounded redox active iron due to the exceed iron buffering capacity of NM may facilitate ferroptotic cell death through mechanisms involving iron-catalyzed lipid peroxidation. In contrast to apoptotic pathways, which were reported in a study by Naoi et al. where NM isolated from post-mortem brain tissues triggered apoptosis in SH-SY5Y cells, our findings revealed that pan-caspase inhibitor Z-VAD-FMK failed to reverse NM-induced cell death.^31^ However, ferroptosis inhibitor ferrostatin-1 successfully rescued cells, supporting the idea that NM-induced toxicity primarily operates through ferroptosis-related mechanisms. Ferroptosis is intricately linked to increased iron levels, lipid peroxidation, decreased GSH levels, and reduced GPX4 activity, all of which are hallmarks of PD pathogenesis.^2,14^ Several in vitro studies have demonstrated that neurotoxins (e.g., 6-OHDA, MPP^+^) induce ferroptosis via lipid peroxidation.^32,33^ Our results suggest a clear distinction in the cell death pathways induced by ifNM and Fe^3+^NM. While ifNM treatment was associated with a combination of autophagy, necrosis, and ferroptosis, Fe^3+^NM primarily may cause ferroptotic cell death. This difference highlights the critical role of iron content in determining NM’s cytotoxic effects and suggests distinct underlying mechanisms in cell death regulation.

### Effects of ifNM and Fe^3+^NM on Lipid Peroxidation

**Figure 6:**
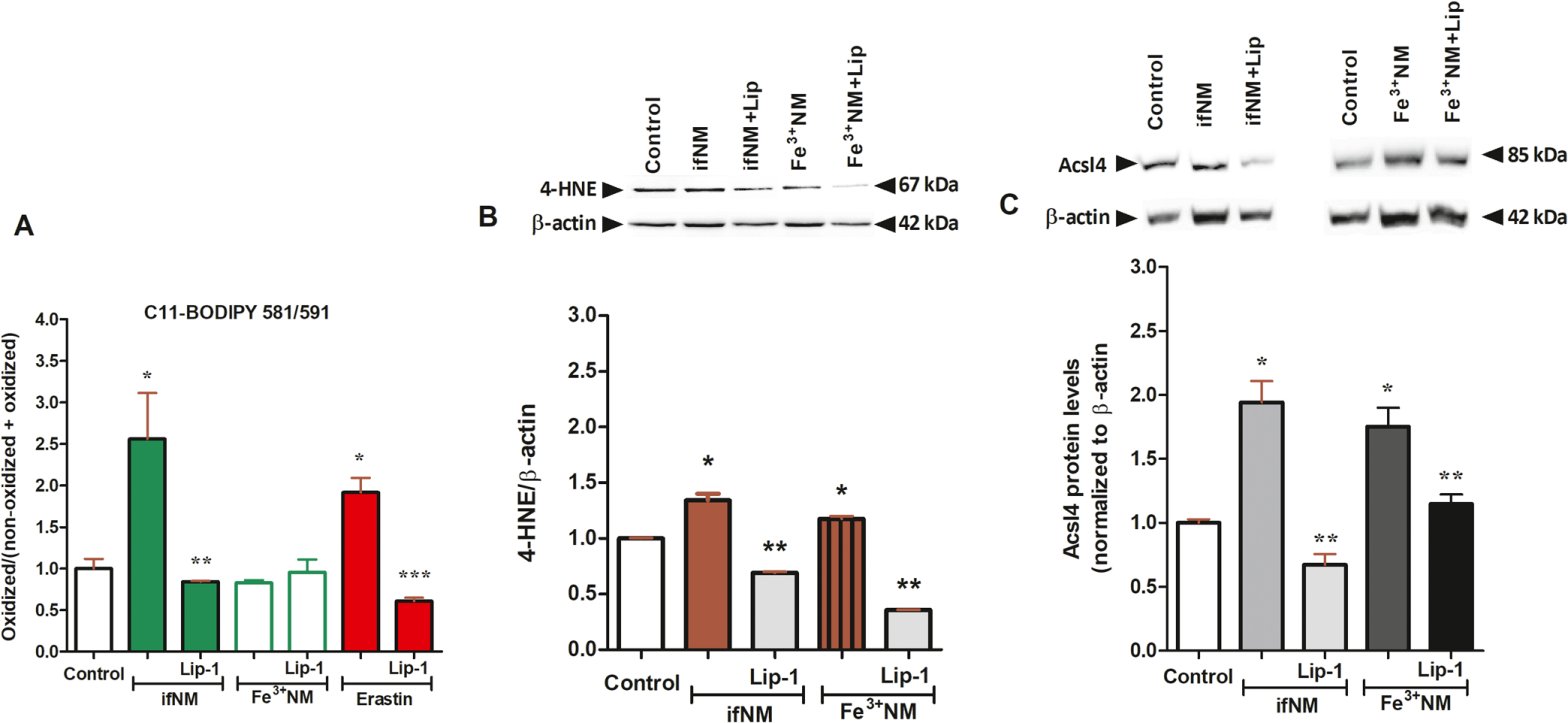
Evaluation of lipid peroxidation in SH-SY5Y cells treated with ifNM and Fe^3+^NM at IC_50_ concentrations through various parameters. Lipid ROS analyzed by BODIPY 581/591 C11 (A), 4-HNE levels (B), and ACSL4 protein expression (C). Protein levels were measured by using Western Blot technique following ifNM (IC_50_), Fe^3+^NM (IC_50_) and ferroptosis inhibitor Liproxstatin-1 (50 nM, 1-h pretreatment) exposure for 24 h. *p < 0.05; compared with vehicle control group, **p < 0.05; compared with only NM-treated groups, ***p < 0.05; compared with only erastin-treated group.

To evaluate the effects of ifNM and Fe^3+^NM on lipid peroxidation, SH-SY5Y cells were exposed to NMs (IC_50_) with or without the lipid peroxidation inhibitor Liproxstatin-1 (50 nM). Lipid ROS levels were assessed using C11-BODIPY 581:591, a hydroperoxide-sensitive fluorescent dye. Fluorescence was significantly higher in ifNM- and Erastin-treated cells compared to the control group, indicating increased lipid peroxidation. Liproxstatin-1 treatment effectively reduced fluorescence in both groups (Figure 6A). Lipid peroxidation was further evaluated through 4-HNE-modified protein levels and ACSL4 protein expression, both of which are well-established markers of ferroptosis. Compared to untreated cells, ifNM and Fe^3+^NM treatments significantly elevated 4-HNE levels (*p* < *0*.05), indicating increased oxidative damage to lipids. This increase was effectively reversed by Liproxstatin-1 treatment in both groups (Figure 6B).

Additionally, both ifNM and Fe^3+^NM treatments upregulated ACSL4 protein expression, further supporting lipid peroxidation-mediated ferroptosis induction. ACSL4 plays a key role in ferroptosis by facilitating the incorporation of polyunsaturated fatty acids (PUFAs) into membrane phospholipids, increasing susceptibility to lipid peroxidation. The observed upregulation of ACSL4 in NM-treated cells suggests that NM may sensitize cells to ferroptosis through enhanced lipid metabolism pathways. Notably, ACSL4 levels were significantly higher in ifNM-treated cells, suggesting a more pronounced activation of ferroptosis-related lipid remodeling in this group. It has been shown that the lipid composition of the cell membrane changes and cells become more sensitive to ferroptosis in ACSL4-overexpressing cells.^34^ However, when cells were pretreated with Liproxstatin-1, ACSL4 expression was significantly reduced in both NM groups (Figure 6C). This suggests that lipid peroxidation is a key driver of ACSL4 upregulation in NM-treated cells and that ferroptosis inhibition can effectively downregulate ACSL4 expression, further supporting the link between NM-induced oxidative stress and ferroptotic cell death.

The consistent decrease in C11-BODIPY fluorescence, 4-HNE levels, and ACSL4 expression upon Liproxstatin-1 treatment highlights a direct relationship between lipid peroxidation, ACSL4-mediated lipid metabolism, and ferroptotic cell death pathways. These findings suggest that NMs trigger ferroptotic cell death through interconnected mechanisms involving lipid ROS accumulation, 4-HNE protein modification, and ACSL4 expression.

### Effects of ifNM and Fe^3+^NM on GSH-GPX4 Axis

**Figure 7:**
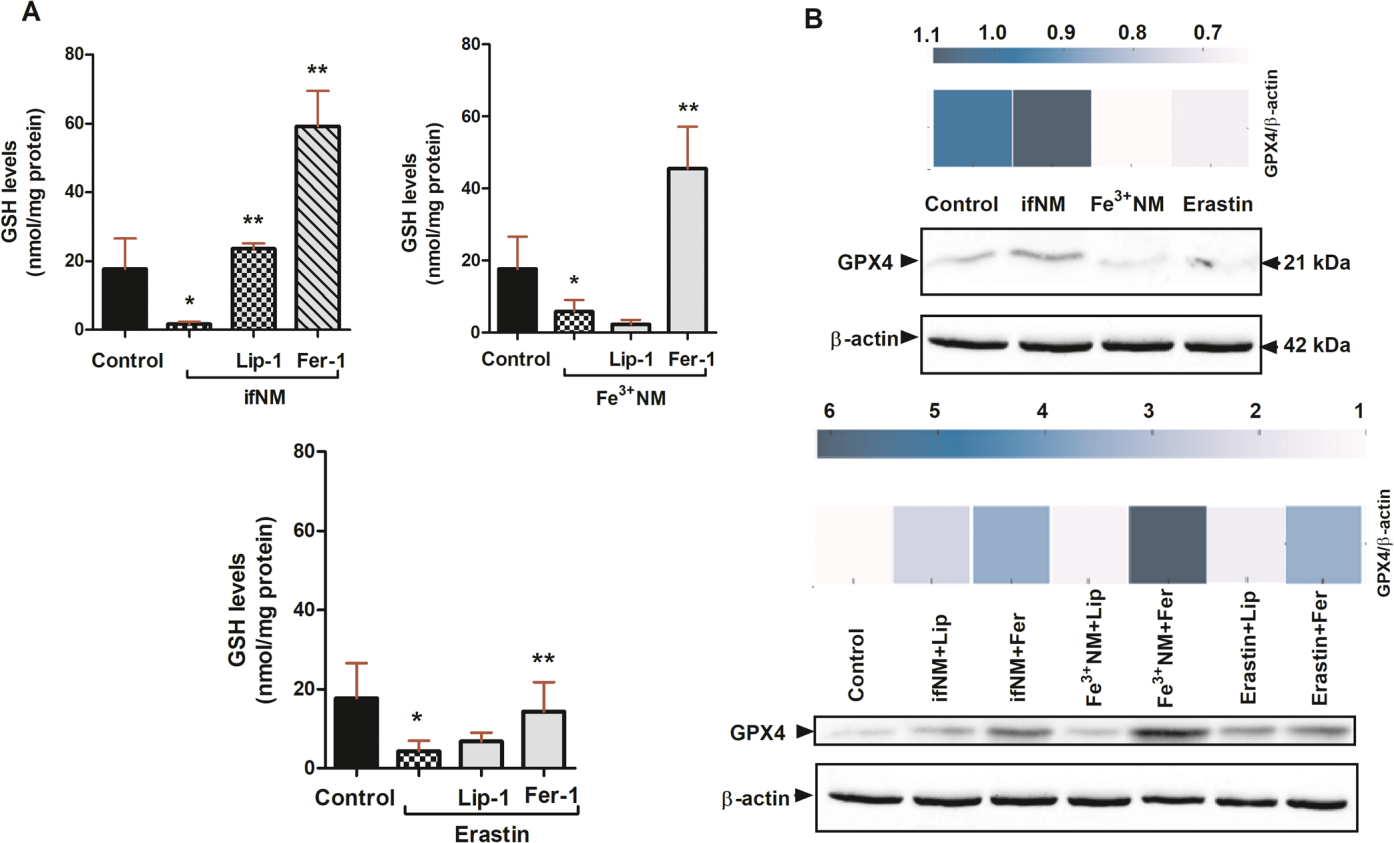
GSH levels in SH-SY5Y cells after 24-h treatments of ifNM (IC_50_), Fe^3+^ NM (IC_50_) and ferroptosis inhibitors (Ferrostatin-1 (5 μM), Liproxstatin-1 (50 nM)). Erastin (30 μM) was used as a ferroptosis inducer (A). Western blot analysis of GPX4 protein expression levels in SH-SY5Y cells under different treatment conditions. Heatmap of the quantitative analysis of GPX4 Western blot results (B). *p < compared with vehicle control group, **p < 0.01; compared with only NM- or erastin-treated cells.

System Xc-, composed of two subunits, a light-chain subunit SLC7A11 (xCT) and a heavy-chain subunit SLC3A2 (CD98, 4F2hc), is a cystine/glutamate antiporter that facilitates the uptake of cystine in exchange for glutamate. An impairment of the system Xc- leads to a depletion in the intracellular cysteine pool, with consequential impairment of GSH biosynthesis and ultimately, GPX4 activity. The subsequent lipid peroxide accumulation results in cell death by ferroptosis.^13^ The study conducted by Bellinger et al. examined the post-mortem brain tissues of patients with and without PD, they encountered significantly reduced GPX4 levels in the tissues of patients with Parkinson’s disease compared to the group without PD.^35^ The fact that GPX4 expression is higher in the SN, compared to other brain regions, makes this enzyme valuable in terms of PD pathogenesis. The measured intracellular GSH levels in SH-SY5Y cells under different treatment conditions revealed significant variations depending on the treatment type and the presence of ferroptosis inhibitors (Ferrostatin-1 and Liproxstatin-1). GSH levels were significantly reduced in both ifNM- and Fe^3+^NM-treated SH-SY5Y cells (Figure 7A). Ferroptosis inhibitors, Ferrostatin-1 and Liproxstatin-1, partially restored GSH levels, with Ferrostatin-1 showing superior efficacy across both ifNM and Fe^3+^NM treatments. Erastin-treated cells showed a marked depletion of GSH, consistent with its role as a ferroptosis inducer. Pretreatment with Ferrostatin-1 and Liproxstatin-1 resulted in a partial recovery of GSH levels, with Ferrostatin-1 being more effective in erastin-treated cells. Erastin-induced GSH depletion was similarly mitigated by these inhibitors, supporting their role in ferroptosis inhibition.

Western blot densitometric analysis revealed a significant decrease in GPX4 levels following both ifNM and Fe^3+^NM treatments, with Fe^3+^NM exhibiting a more pronounced reduction. Pretreatment with Ferrostatin-1 and Liproxstatin-1 partially restored GPX4 levels, with Ferrostatin-1 demonstrating superior protective effects (Figure 7B). These findings suggest that GPX4 depletion is a key event in NM-induced ferroptosis, with distinct patterns observed between ifNM and Fe^3+^NM treatments.

### Intracellular Iron (Fe^2+^) and Iron Metabolism-Related Protein Alterations Induced by ifNM and Fe^3+^NM

Iron metabolism plays a crucial role in maintaining cellular homeostasis, and its dysregulation is a key driver of ferroptosis.^14^ Transferrin receptor 1 (TfR1) facilitates iron uptake by mediating the internalization of transferrin-bound iron into cells through endocytosis. Once inside the cell, iron is released from transferrin and converted into ferrous iron (Fe^2+^) within the endosome before being transported into the cytoplasm via divalent metal transporter 1 (DMT1). The ability of iron to promote ferroptosis primarily depends on its ferrous (Fe^2+^) state, which participates in Fenton chemistry, generating reactive oxygen species.^30^ Under physiological conditions, iron exists in two oxidation states—ferrous (Fe^2+^) and ferric (Fe^3+^)—which are tightly regulated to prevent toxicity.^36^

**Figure 8:**
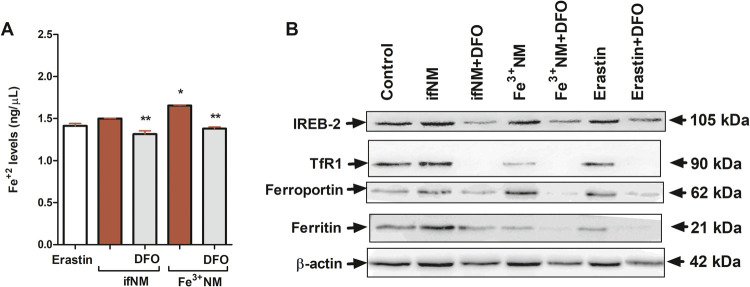
Fe^2+^ levels (A) and iron metabolism-related protein expression levels (B) in SH-SY5Y cells following ifNM (IC_50_) and Fe^3+^NM (IC_50_) treatments, with or without iron chelator DFO (100 μM, 1 h pretreatment) for 24 h. The expression of IREB-2, TfR1, ferroportin and ferritin in cells were determined by Western blot analysis. *p < 0.05; compared with vehicle control group, **p < 0.05; compared with only NM-treated cells.

Ferroportin (FPN) is the only known iron exporter in mammalian cells, responsible for transporting excess iron out of the cell. A decrease in ferroportin leads to intracellular iron accumulation, enhancing susceptibility to ferroptosis. In our study, intracellular Fe^2+^ levels significantly increased following Fe^3+^NM treatment compared to the control group (*p* < 0.05) (Figure 8A). Additionally, Fe^3+^NM treatment significantly upregulated TfR1 levels, an effect reversed by deferoxamine (DFO) pretreatment, highlighting a feedback mechanism in response to altered iron homeostasis. Western blot analysis revealed slight elevation in ferroportin levels following Fe^3+^NM (1.56-fold) and Erastin (1.40-fold) treatments (*p* < 0.01), suggesting an adaptive response to oxidative stress-induced iron efflux (Figure 8B). DFO, a widely used iron chelator, effectively reduced ferroportin levels in the ifNM + DFO (*p* < 0.05), Fe^3+^NM + DFO (*p* < 0.01), and Erastin + DFO (*p* < 0.05) groups.

To prevent iron toxicity, excess intracellular iron is stored within ferritin, a cytosolic iron-storage protein that sequesters free iron in a redox-inactive form. Under conditions of iron overload or oxidative stress, ferritin can undergo ferritinophagy, leading to iron release into the labile iron pool, fueling Fenton chemistry and ROS production. In our study, ferritin levels increased following ifNM treatment, reflecting an adaptive response to iron overload. In contrast, Fe^3+^NM and Erastin treatments led to ferritin depletion, possibly through ferritinophagy, which may exacerbate oxidative stress. DFO pretreatment partially restored ferritin levels in Fe^3+^NM and Erastin groups but significantly reduced them in ifNM-treated cells, suggesting a complex regulatory interaction between NM and intracellular iron storage mechanisms.

Iron responsive element binding protein 2 (IREB2) is a critical sensor of intracellular iron levels, regulating iron metabolism-related proteins. IREB2 controls iron availability by modulating ferritin and TfR1 expression based on cellular iron demand. Studies have demonstrated that IREB2 is essential for ferroptosis, as it enhances iron uptake while suppressing iron storage, thereby promoting iron-dependent lipid peroxidation. In our study, IREB2 levels increased following ifNM, Fe^3+^NM, and Erastin treatments, indicating activation of iron regulatory pathways under oxidative stress. However, DFO pretreatment significantly reduced IREB2 levels across all groups, suggesting a compensatory regulatory mechanism via iron chelation.

While our study focuses on the ferroptosis-inducing effects of NM in dopaminergic SH-SY5Y cells, these findings have potential in vivo implications, particularly in the context of PD models. Post-mortem analyses of PD patient brains have demonstrated increased iron deposition in the SNpc, accompanied by elevated lipid peroxidation markers, which align with our in vitro findings. Future studies using in vivo models of PD could further elucidate the interplay between NM accumulation, iron homeostasis, and ferroptosis in neurodegeneration. Investigating whether ferroptosis inhibitors (e.g., Liproxstatin-1, Ferrostatin-1) can protect NM-rich neurons from ferroptotic cell death in animal models would provide crucial translational insights. Additionally, assessing the role of NM-bound iron in ferroptosis-related gene expression and neuronal viability in PD animal models could bridge the gap between our in vitro observations and potential disease-modifying strategies.

## Conclusions

In summary, our findings collectively highlight the complex interplay between iron metabolism, oxidative stress, and ferroptosis pathways in response to NM treatments. While this study provides valuable insights into the relationship between neuromelanin and ferroptosis, some limitations warrant consideration. First, the synthetic NM pigments (ifNM and Fe^3+^NM) used in this study, although chemically and structurally analogous to endogenous NM, may not perfectly mimic the biochemical properties of NM in the human brain. Refining synthesis methods to better replicate endogenous NM could strengthen the translational relevance of these findings. Additionally, future research could integrate multiomics approaches to unravel the broader impact of NM on cellular pathways. These limitations highlight the need for complementary in vivo studies to better understand NM’s role in neuronal health and its potential as a therapeutic target in neurodegeneration. Future research should also explore the development of ferroptosis inhibitors and iron-chelating agents as promising therapeutic strategies for mitigating NM-induced ferroptosis and oxidative stress.

## Materials and Methods

### Preparation of Iron-Free (ifNM) and Iron-Containing (Fe^3+^NM) NMs

ifNM preparation: Dopamine (Cayman Chemicals) (1.5 g) and cysteine (Sigma) (232 mg) were dissolved in 1000 mL of 50 mM phosphate buffer (Thermo Fisher Scientific, pH 7.4) at a molar ratio of 6:1. The solution was incubated at 37 °C in an orbital shaker with free air access for 3 days. The resulting black mixture was collected by centrifugation at 10,000 rpm for 10 min and washed once with 1% acetic acid solution (CARLO ERBA Reagents, France) and twice with distilled water. Finally, NM was dried under vacuum and kept at +4 °C until the day of the experiment.^17^

Fe^3+^NM preparation: NM was prepared as described as ifNM. FeCl_3_·6H_2_O (Sigma) was added to the mixture at a final concentration of 1 mM following the 3-day incubation period.^37^

### Characterization of Synthetic NMs

Scanning Electron Microscopy (SEM): NM samples were prepared as described above. Silicon wafers containing dried NM film were mounted on stainless steel pegs using double-rod copper tape before being transferred to the SEM chamber. Mounted samples were coated with gold–palladium mist under argon plasma for 4 min at 10 mA using a Hummer V sputter coater (Anatech, Springfield, VA). A Philips FEI XL30 SEM-FEG (FEI, Portland, OR) equipped with a backscatter secondary electron detector and a resolution of 3 nm was used to examine the samples in ultrahigh resolution mode. Typical electron beam conditions were 3–5 kV with 3.0 spot size and 4.0–6.0 mm working distance. SEM images were acquired and analyzed using analySIS XL docu software (Soft Imaging Systems, Lakewood, CO). All images were saved as TIFF files.^20^

Infrared (IR) Spectroscopy: The structures of NM samples were confirmed by IR spectra and recorded as potassium bromide pellets on a Jasco FT/IR-400 spectrometer (Jasco, Tokyo, Japan).^38^

### Cell Culture Studies and Treatments

Human neuroblastoma SH-SY5Y cells (ATCC CRL-2266) were cultured in DMEM/F12 medium supplemented with 10% fetal bovine serum, 2 mM l-glutamine, and 100 U/mL penicillin-streptomycin. Cells were incubated at 37 °C in a 5% CO_2_ and passaged at a 1:4 ratio, with passages limited to 20. After reaching the desired confluency, cells were detached using 0.25% trypsin, stained with trypan blue, and counted using a hemocytometer. For experiments, cells were seeded at a density of 0.5 × 10^6^ cells/well in 6-well plates or 2 × 10^3^ cells/well in 96-well plates. Following the adherence of the cells, lyophilized synthetic NMs were weighed, suspended in culture medium at 10X the highest intended concentration (main stock), and serially diluted to the desired concentrations. Before application, suspensions were vortexed to ensure even pigment distribution and prevent precipitation. Cells were pretreated for 1 or 2 h with apoptosis (Z-VAD-FMK, 20 μM), necrosis (necrostatin-1, 50 μM), autophagy (3-methyladenine, 3-MA, 5 mM), ferroptosis (Ferrostatin-1, 5 μM; Liproxstatin-1, 50 nM) inhibitors, *N*-acetylcysteine (NAC, 1 mM), Trolox (100 μM), and deferoxamine (DFO, 100 μM) followed by 24-h NM (IC_50_) exposure. Erastin (30 μM) was used as a positive control to induce ferroptosis. Cells treated with DMSO (0.1%) were used as vehicle control

### Cell Entry of Synthetic NM

After 48 h of incubation, the cells were visualized with a light microscope to verify the entry of NMs into the cell.

### WST-1 Assay

Cell viability was assessed using the WST-1 assay (Takara Bio, MK400, Japan). Cells were exposed to six NM concentrations (1, 10, 25, 50, 75, and 100 μg/mL) for 24 and 48 h to determine IC_50_ values. WST-1 solution (20 μL) was added, incubated for 3 h, and absorbance was measured at 450 nm using a microplate reader (Thermo Fisher Scientific). Cell survival was normalized to untreated cells.

### LDH Assay

LDH release was measured using the LDH assay kit (Cayman Chemicals, 601170) following the manufacturer’s protocol. After 24- and 48-h NM application, 50 μL of the medium was mixed with 50 μL of LDH reaction mixture and incubated for 25 min at room temperature. Optical density was measured at a wavelength of 492 nm/690 nm with a microplate reader. Rotenone (1 and 10 μM) was used to compare LDH release. 10% Triton X-100-treated wells were used as maximum release (100%) wells.

### Protein Analysis

After treatments, cells were washed with ice-cold PBS and lysed in 1X cell lysis buffer containing phosphatase and protease inhibitors. Protein concentrations were determined using the BCA assay (Thermo Fisher Scientific). Equal amounts of protein (30 μg) were separated on 10% SDS-PAGE gels and transferred to PVDF membranes. Membranes were blocked and incubated overnight at +4 °C with primary antibodies: anti-4-HNE (1:1000), anti-GPX4 (1:500), anti-Acsl-4 (1:500), anti-TfR-1 (1:1000), anti-Ferroportin (1:1000), anti-IREB-2 (1:1000), and anti-Ferritin (1:1000). After washing, membranes were incubated with HRP-conjugated secondary antibodies (1:1000, Cell Signaling Technology, USA) for 1 h at room temperature. β-actin (1:1000) was used as a loading control. Protein bands were detected using chemiluminescence reagents (Millipore Sigma) and visualized with a Fusion Fx7 imager. Densitometric analysis was performed using ImageJ software (NIH). Experiments were repeated at least twice.

### GSH Assay

GSH levels were measured using a GSH assay kit (Elabsciences, E-BC–K030-S) following the manufacturer’s protocol. After NM treatment, cells were homogenized in lysis buffer, and homogenates were centrifuged at 4500*g* for 10 min. Supernatants were collected, mixed with assay reagents, and incubated for 15 min at room temperature. Absorbance was measured colorimetrically at 420 nm using a microplate reader. GSH levels were expressed as mg/g protein.

### Detection of Lipid ROS by C11-BODIPY 581:591

Lipid hydroperoxide levels were assessed using the fluorescent dye C11-BODIPY 581:591 (Cayman Chemical, 27086) following NM treatments. Erastin (30 μM) served as a positive control, while Liproxstatin-1 (50 nM) was used as a ferroptosis inhibitor. After 24 h of exposure, the medium was replaced with fresh medium containing 2 μM C11-BODIPY 581/591 and incubated at 37 °C for 30 min (Liu et al.). The rates of lipid peroxidation were measured using a Varioskan Flash multimode reader (Thermo Fisher Scientific) with excitation/emission of 495/521 nm (oxidized) and 575:600 nm (nonoxidized dye). Oxidation of the dye was expressed as a ratio between oxidized and total oxidized + nonoxidized forms.

### Iron Assay

Intracellular Fe^2+^ levels were measured using an iron assay kit (Sigma, MAK025) following the manufacturer’s protocol. After NM treatment, cells were homogenized in lysis buffer, and 10 μL of cell homogenate was transferred to 96-well plates. The volume was adjusted to 100 μL with measuring buffer and incubated at room temperature for 30 min, protected from light. Next, 100 μL of Iron Probe solution was added, and plates were incubated for an additional 60 min at room temperature, protected from light. Absorbance was measured colorimetrically at 593 nm using a microplate reader.

### Statistical Analysis

The data are presented as mean ± standard deviation (SD). Each group was analyzed in at least three independent experiments, with each experiment performed in triplicate. Statistical analyses were conducted using GraphPad Prism 4.03 for Windows (GraphPad Software, San Diego, CA). One-way analysis of variance (ANOVA) followed by Tukey’s post hoc test was used for multiple group comparisons, while two-way ANOVA was applied to evaluate overall significance across groups. Statistical significance was determined at *p* < 0.05, with Bonferroni correction applied for multiple comparisons.

## Notes

Declaration of generative AI in scientific writing: during the preparation of this work, the author(s) used ChatGPT4o in order to enhance readability and text understanding. After using this tool/service, the author(s) reviewed and edited the content as needed and took full responsibility for the content of the publication.

## Abbreviations

3-MA: 3-methyladenine
4-HNE: 4-hydroxynonenal
Acsl-4: acyl-CoA synthetase long-chain family member 4
Cys: cysteine
DFO: deferoxamine
DMT1: divalent metal transporter 1
Fe^3+^NM: iron containing neuromelanin
GPX4: glutathione peroxidase 4
GSH: glutathione
IREB2: iron responsive element binding protein 2
ifNM: iron free neuromelanin
NM: neuromelanin
NAC: *N*-acetylcysteine
ROS: reactive oxygen species
RSL-3: RAS-selective lethal 3
SH-SY5Y: human neuroblastoma cell line
SNPc: substantia Nigra Pars compacta
TfR1: transferrin receptor 1
Z-VAD-FMK: Pan-caspase inhibitor
